# Can the gut-brain axis provide insight into psilocybin's therapeutic value in reducing stress?

**DOI:** 10.1016/j.ynstr.2025.100732

**Published:** 2025-05-19

**Authors:** Alanna Kit, Kate Conway, Savannah Makarowski, Grace O'Regan, Josh Allen, Sandy R. Shultz, Tamara S. Bodnar, Brian R. Christie

**Affiliations:** a Division of Medical Sciences, University of Victoria, Victoria, BC, Canada; b Island Medical Program, University of British Columbia, Victoria, BC, Canada; c Centre for Trauma & Mental Health Research, Vancouver Island University, Nanaimo, BC, Canada; d Department of Neuroscience, School of Translational Medicine, Monash University, Melbourne, VIC, Australia; e Department of Biological Sciences, University of Calgary, Calgary, Alberta, Canada; f Institute for Aging and Lifelong Health, University of Victoria, 3800 Finnerty Rd, Victoria, BC, V8P 5C2, Canada; g Center for Behavioral Teratology, San Diego State University, 6330 Alvarado Ct., San Diego, CA, 92120, USA

**Keywords:** Psychedelics, Hallucinogens, Microbiome, Inflammation, 5-HT, Neuropsychiatric disorders

## Abstract

There is growing interest in exploring the therapeutic potential and mechanisms of action of psilocybin on stress-related neuropsychiatric disorders, including depression, generalized anxiety disorder (GAD), post-traumatic stress disorder (PTSD), obsessive-compulsive disorder (OCD), addiction, and disordered eating. Despite promising progressions in preclinical and clinical research, the neurobiological and physiological mechanisms underlying the therapeutic effects of psilocybin remain complex, involving multiple systems with numerous homeostatic feedback signaling pathways throughout the body. This review paper explores how psilocybin mechanistically interacts with the gut microbiota, enteric nervous system, hypothalamic-pituitary axis, and how psilocybin influences the bidirectional communication between peripheral and neuronal systems. Shifting towards a more integrated paradigm to unravel the mechanisms through which psilocybin affects the bidirectional gut-brain axis holds the promise of significantly advancing our understanding of psilocybin-based therapies from preparation of treatment, administration, to proceeding long-term integration. Such an understanding can extend beyond the treatment of psychiatric disorders, further encompassing a broader spectrum of inflammatory-related disorders.

## List of abbreviations:

5-HT: 5-hydroxytryptamine
5-HT_2A/2B/2C_: 5-HT receptor subtype _2A/2B/2C_
BBB: Blood-Brain Barrier
BDNF: brain-derived neurotrophic factor
CeA: Central amygdala
CNS: central nervous system
CREB: (cAMP)-response element binding protein
ENS: Enteric nervous system
GAD: Generalized anxiety disorder
GBA: gut-brain axis
GI: gastrointestinal
HPA: hypothalamic pituitary axis
HTR: head twitch response
Kyn: kynurenine
IL: Interleukin
MDD: major depressive disorder
NFAT: nuclear factor of activated T cells
Nf-kB: nuclear factor κB
OCD: obsessive compulsive disorder
PNS: peripheral nervous system
ROS: reactive oxygen species
PTSD: Post traumatic stress disorder
SSRI: selective serotonin reuptake inhibitor
Trp: Tryptophan

## 1 A brief history of psilocybin

The Fungi kingdom, which encompasses all species of mushrooms, is approximately 2.4 billion years old, and has entangled relationships throughout the ecological pyramid (Bengtson et al., 2017). There are currently 200 known varieties of mushrooms that contain psilocybin (Rodríguez Arce and Winkelman, 2021). Historically, psilocybin containing mushrooms have been actively used in ceremonial ritualistic practices among Indigenous peoples and cultures around most parts of the world for several millennia (Carod-Artal, 2015). In the 1950's, government sponsored research first began to explore the therapeutic potential of psilocybin, among other psychedelic compounds (Tupper et al., 2015). Despite initial successes in several areas, ranging from depression to palliative care, the Comprehensive Drug Abuse Control and Prevention Act was introduced in the US in 1970, and this brought mainstream psychedelic research to an abrupt stand still worldwide until the early 2000s. Over the past two decades, psilocybin has become more widely accepted as a promising therapy for treatment-resistant and major depressive disorder, as well as end-of-life distress in terminal patients (Carhart-Harris et al., 2016; Griffiths et al., 2016; Ross et al., 2016). Preclinical and clinical studies demonstrate that psilocybin produces antidepressant, anxiolytic, and anti-addictive properties following a single high-dose treatment, with sustained benefits for up to 6–18 months, due to a combination of anecdotal and neurobiological mechanisms (de Vos et al., 2021; Flanagan and Nichols, 2018; Rifkin et al., 2020).

### 1.1 Metabolism of psilocybin

**Fig. 1:**
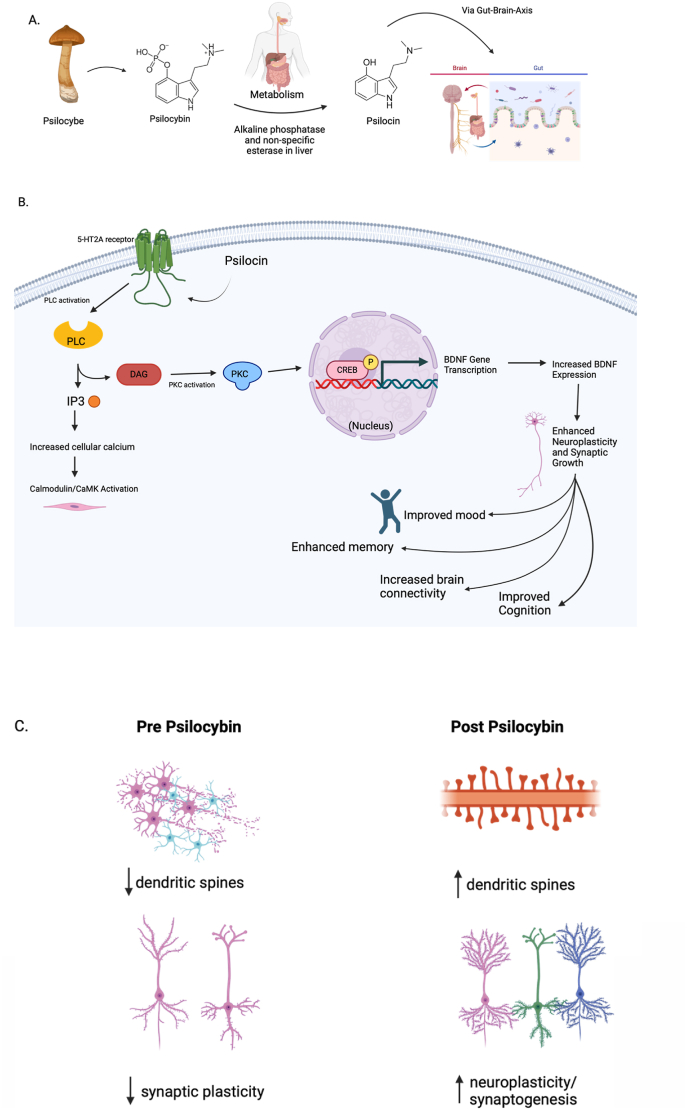
**Properties of psilocybin and 5-HT_2A_ receptors.** A) metabolism of psilocybin. After oral ingestion, psilocybin is rapidly absorbed from the GI tract into the bloodstream. Psilocybin is converted into its active form, psilocin, primarily in the liver, through the action of alkaline phosphatase enzymes. Psilocin is distributed throughout the body, with high concentrations reaching the brain, where it crosses the blood-brain barrier and exerts its effects. B) Psilocin binds to 5-HT_2A_ receptors, leading to the activation of phospholipase C (PLC) and resulting in the generation of inositol trisphosphate (IP3) leading to increased intracellular calcium, and diacylglycerol (DAG). This triggers activation of protein kinase C (PKC). These signals subsequently phosphorylate transcription factors such as CREB (cAMP response element-binding protein). CREB binds to the BDNF gene promoter, enhancing BDNF transcription and leading to increased BDNF protein levels. Increased BDNF supports neuroplasticity, which promote the survival and growth of neurons, enhancing synaptic plasticity, and supporting cognitive functions. C) The combination of increased intracellular signaling and BDNF support results in the growth and formation of new dendritic spines. These new spines increase the surface area available for synaptic connections, enhancing synaptic plasticity. Increased synaptic plasticity promotes the survival and growth of neurons, enhancing synaptic plasticity, and supporting cognitive functions. In contrast, dendritic spines are susceptible to degenerating and losing connections, observed in oxidative stress, neuroinflammation, and altered neurotrophic support.

Psilocybin is a tryptamine derivative and naturally occurring serotonergic psychedelic produced by numerous species of mushrooms primarily of the *Psilocybe* genus (Passie et al., 2002). It's active form psilocin shares similarity in structure to the key pleiotropic neurotransmitter serotonin (5-HT), due to its indole ring (Fig. 1A) (Dinis-Oliveira, 2017). Preclinical studies have shown that when psilocybin is administered orally to rodents, it is dephosphorylated into psilocin in the intestinal mucosa by alkaline phosphatase and esterase with 50 % of the psilocin absorbed from the digestive track (Dinis-Oliveira, 2017). Therefore, it is also important to consider factors such as route of psilocybin administration in preclinical and clinical research (i.e., intravenous, intraperitoneal, and oral). Interestingly, majority of preclinical psilocybin research in rodents has used intraperitoneal (IP) administration to study the pharmacology, toxicity, and mechanisms of action of psilocybin (Pedicini and Cordner, 2023). This has the advantage of there being rapid and reliable absorption of psilocybin in targeted tissues, such as the brain, while avoiding gastrointestinal variables, but does not guarantee full involvement of the GBA system. While reports indicate that the therapeutic effects of a single high-dose psilocybin treatment are correlated to the intensity of the psychedelic experience, it is possible that low doses may also indirectly act on the CNS through a more sustained interaction with the gut microbiota, given the consistent low dosing regimen and the bidirectional communication pathway of the gut and brain (Kuypers, 2019). Given that there may be an important role for the GBA for some of psilocybin's effects, more research involving oral administration of psilocybin is required. In addition, this would allow for a better translation of mechanistic preclinical conclusions into clinical populations.

In clinical settings, and in recreational use, psilocybin is normally orally administered, travels through the gastrointestinal system where it is further metabolized/dephosphorylated into psilocin and then absorbed into the bloodstream where it is further filtered through the liver via the hepatic portal vein (Dinis-Oliveira, 2017) (Fig. 1A). Considering that approximately 90–95 % of the total body's 5-HT is synthesized in the enterochromaffin (EC) cells of the GI tract (Han et al., 2022b; Terry and Margolis, 2017), it is reasonable to expect that there may be an interaction of psilocybin with the microbiota gut-brain pathway (Kuypers, 2019). However, the majority of existing psychedelic research has focused primarily on the impact and mechanism of action of psilocybin in the CNS (Cardeña and Lynn, 2020; Ling et al., 2022; Rifkin et al., 2020).

## 2 5-HT_2A_ receptors and psilocybin

5-HT is a monoamine neurotransmitter that influences sleep, appetite, memory, learning, temperature, sexual behaviour, pain sensation, and inflammation (Shajib et al., 2017; Yano et al., 2015). Psilocin agonizes a variety of 5-HT receptors in the peripheral nervous system (PNS) and central nervous system (CNS), with its primary psychoactive effects mediated by the 5-HT_2A_ receptor (Carhart-Harris et al., 2012; Nichols, 2004).

The 5-HT_2A_ receptor is a G_(q)_ protein-coupled receptor subtype of the 5-HT receptor family that plays a significant role in the central nervous system's regulation of mood, perception, and cognition through several intracellular signaling pathways including mitogen-activated protein kinase (MAPK) pathway and protein kinase B (PKB/Akt) pathway (Zhang and Stackman, 2015). Activation of the 5-HT_2A_ receptor stimulates the MAPK pathway, which then leads to changes in gene expression and cellular responses related to growth and differentiation as well as the PKB/Akt pathway, which is a process involved in cell survival and growth (Fig. 1B) (Yu et al., 2008). These pathways contribute to the complex physiological and behavioural effects, including neurogenic and antidepressant-like responses (Fig. 1C) (de Vos et al., 2021; Vargas et al., 2023). Given that psilocybin is a 5-HT receptor agonist, it is important to note that the majority of 5-HT_2A_ receptors are found in the CNS and in the smooth muscle cells of the gut vasculature (Beattie et al., 2004). Expression of the 5-HT_2A_ receptors in the CNS is most abundant in the cortex, hippocampus, basal ganglia, and forebrain (Liu et al., 2020; Zhang and Stackman, 2015). More specifically to the CNS, 5-HT_2A_ receptors are also abundantly expressed on neocortical GABAergic interneurons and layer V pyramidal neurons, the latter of which have dendrites that span all cortical layers (Scott and Carhart-Harris, 2019). 5-HT_2A_ receptors modulate the release of several neurotransmitters, such as glutamate, GABA, and dopamine, and this modulation is critical in maintaining the balance of excitatory and inhibitory signals for proper signal transduction (Allen et al., 2024). One review summarized that 5-HT_2A_ receptors in the gut are highly expressed on both enteric neurons and gut epithelial cells, where they control smooth muscle contraction and regulate growth factors, paracrine factors, hormones, and neurotransmitters (Jedlitschky et al., 2012). As such, psilocybin-induced 5-HT_2A_ receptor activity leads to network-level brain changes that correlate with the intensity of the experience, including dissolution of the ego – the transient merging of self-boundaries with the external world – and the magnitude of therapeutic responsiveness (Girn et al., 2023; Griffiths et al., 2011; Luppi et al., 2023).

The therapeutic effects of psilocybin are associated with the reopening of a window of increased neuroplasticity, wherein the brain is more malleable than usual and sensitive to novel experiences (Nardou et al., 2023). Evidence suggests that psilocybin induces rapid and persistent 5-HT_2A_-dependent increases in neuritogenesis (Ly et al., 2018; Shao et al., 2021) and a strengthening of synapses (Hesselgrave et al., 2021). Interestingly, a recent study reported that a large proportion of 5-HT_2A_ receptors in cortical neurons are localized intracellularly, rather than extracellularly (Vargas et al., 2023). Unlike 5-HT, psilocybin exhibits highly lipophilic properties, enabling it to traverse the neuronal cell membrane and activate intracellular 5-HT_2A_ receptors (Vargas et al., 2023). Researchers have determined that the activation of these receptors is essential for the neurogenic effects of psychedelics and the subsequent antidepressant-like behavioural responses (Vargas et al., 2023). To understand the mechanisms of how 5-HT_2A_ receptors can contribute to neuroplasticity, it is first necessary to examine the receptor activation and biosynthesis pathway (Nardou et al., 2023).

## 3 Psilocybin and the gut microbiome

Psilocybin is normally ingested orally, and thus it has direct effects on the gut-brain axis (GBA), also known as gut-brain connection or microbiota-gut-brain axis. It is a bidirectional communication pathway linking the enteric nervous system (ENS) and the central nervous system (CNS) (Mayer, 2011). This axis involves a range of interconnected bidirectional systems and metabolites, including the nervous, endocrine, and immune systems, the bacteria contained within the gut (gut microbiota), its gene products, metabolites, and signaling molecules, all participating in mutual regulation and homeostatic functions (Cussotto et al., 2018; Martin et al., 2018). The vagus nerve (cranial nerve X) originates in the brainstem, specifically the medulla oblongata. It is the longest mixed nerve (i.e., containing both afferent and efferent fibres) of the ANS and a key route of communication between the ENS and the brain, playing a fundamental role in interoceptive awareness and homeostatic function (Bonaz et al., 2018; Fülling et al., 2019; Liu et al., 2020). This cranial nerve innervates visceral organs and is composed of about 80 % afferent and 20 % efferent nerves, with data suggesting that it can have anti-inflammatory effects due to its role in the cholinergic anti-inflammatory pathway (Bonaz et al., 2016). Vagal visceral afferent nerve fiber divisions can detect microbial metabolites, such as short-chain fatty acids (SCFAs), presence of cytokines and neurotransmitters associated with microbial composition. These signals are transmitted to the brainstem (nucleus tractus solitarius), triggering a coordinated response to modulate inflammation (Bonaz et al., 2018; Cryan et al., 2019; Fülling et al., 2019; Han et al., 2022a; Ma et al., 2019). Vagal nerve stimulation is a well-established antidepressant treatment used in meditation, breathwork, and other practices (Gerritsen and Band, 2018). Specific gut bacteria have also been shown to either produce or stimulate the production of several neuroactive molecules, such as 5-HT, which activate the ENS and vagus nerve, thus influencing brain function and associated behaviour (Cryan et al., 2019; Cryan and Dinan, 2012). One systematic review proposed a novel perspective on central serotonin activity, suggesting it functions as a “rostral extension” of the enteric serotonergic system, modulated via hypothalamic control over descending serotonergic nuclei in the brainstem (Shine et al., 2022). This framework posits that serotonergic tone enables brain-wide cognitive flexibility by enabling dynamic switching between cognitive modes. Acting as a metaplastic neuromodulator of cortical and subcortical circuits, serotonin helps regulate the balance between habitual and adaptive behaviours. The authors further propose that psychedelics potentiate this serotonergic system by amplifying serotonergic mode of cognition: one that favours flexibility, enhanced associative thinking, and openness to novel experiences (Shine et al., 2022).

Bacteria within the gut produce a diverse number of molecules that influence the Hypothalamic Pituitary Axis (HPA) by activation of the ENS, vagus nerve, and possibly directly by targeting receptors in brain regions including the hypothalamus by reach of the blood-brain barrier (BBB) through the ventricular space (Cussotto et al., 2018; Latorre et al., 2016). As a result, certain SCFAs have been shown to impact our mood, stress, and immune responses, by directly or indirectly communicating with the brain and modulating the regulation of neuroplasticity, epigenetics, and gene expression, thereby influencing CNS adaptation within certain brain regions (Han et al., 2022a). For example, SCFAs, such as butyrate, propionate, and acetate can cross the blood-brain barrier (BBB) and influence neurons and glial cells, to regulate central nervous system (CNS) functions, via activation of G-protein-coupled receptors (GPR41 and GPR43), which can modulate neuronal signaling (O'Riordan et al., 2022). Considering indirect chemical signaling from the afferent pathway of the vagus nerve, gut microbiota can also modulate and influence various aspects of brain function and neurotransmitters, such as 5-HT, dopamine, norepinephrine, and GABA (Ma et al., 2019). This modulation plays a crucial role in the regulation of mood, stress, emotion, cognition, memory, and learning (Cryan et al., 2019; Huang and Wu, 2021; Yano et al., 2015), by acting either directly on the vagal afferent receptors or indirectly on enteric neurons, innervating the gut epithelial lining (Fülling et al., 2019). Microbial metabolites, such as SCFAs, particularly butyrate, stimulate neurotrophic production of brain-derived neurotrophic factor (BDNF) in the CNS, by crossing the BBB and promoting upregulation of BDNF expression in the hippocampus, a key brain region for learning, memory, and mood (Cryan et al., 2019; Cryan and Dinan, 2012; Fülling et al., 2019). Interestingly, BDNF binds to TrkB receptors, which are allosterically modulated with high affinity by psilocybin and LSD (Moliner et al., 2023). The gut metabolites regulate the metabolism of the amino acid tryptophan into serotonin, kynurenine, and other metabolites, which can impact brain signaling by upregulating BDNF levels (Bosi et al., 2020). Moreover, the human microbiota not only guards the host from external pathogens through production of antimicrobial substances but is also a significant constituent in the generation of intestinal mucosal layer, immune system maturation and immune homeostasis (Cryan et al., 2019; Shajib et al., 2017). When in balance, they are crucial modulators to systemic inflammation by supressing pro-inflammatory cytokines, such as TNF-α and IL-1β by inhibiting inflammatory signaling pathways like NF-κB, while enhancing anti-inflammatory cytokines, such as IL-10, produced by regulatory immune T cells (Tregs) and macrophages (Zhao et al., 2023). When out of balance, the gut microbiota can lead to intestinal barrier dysfunction, which can increase the body's susceptibility to infections, autoimmune reactions, and inflammatory related diseases. For example, a study conducted chronic feeding with lactic acid bacteria *Lactobacillus rhamnosus* strain on mice and discovered region-dependent modifications in the brain, such as upregulation and expression of the GABA receptor, an inhibitory neurotransmitter, in cortical regions along with the hippocampus, amygdala, and *locus coeruleus* (Bravo et al., 2011). This further demonstrates how the balance of gut microbiota is able to modulate and influence neurophysiology and behaviour. Therefore, both the gut microbiota and immune system have been tightly associated with the manifestation of etiopathogenesis or initiation of neurodevelopmental, psychiatric, and neurodegenerative diseases (Rudzki and Maes, 2020). Perturbation of this vital communication between gut microbiota, EECs, ENS and central nervous system may affect both physiological and homeostatic gut functionality (Latorre et al., 2016).

## 4 The hypothalamic-pituitary-axis (HPA)

The HPA axis is a major neuroendocrine pathway that links perceived stress with physiological and behavioural coping reactions (Rusch et al., 2023). Brain regions such as the hypothalamus, medial prefrontal cortex, and central amygdala (CeA) receive and integrate sensory information from organs and tissues, which then generate output signals to modify physiological responses, whilst working with the adrenal and pituitary glands to maintain homeostatic states (Smith and Vale, 2006). It is also important to note that the neuro-peptide nuclei within the hypothalamus are a crucial point of convergence for neuro-endocrine-immune responses and the development of depression (Bao and Swaab, 2019). The HPA axis serves as a critical modulator in response to perceived stress and inflammation, while also interacting with the neuroendocrine and immune systems' responses to stress, influencing mood through elevated levels of cortisol concentration and corticotropin-releasing hormone (CRH). (Bao and Swaab, 2019). The interconnectivity of the gut-brain-endocrine-immune systems reflects a highly integrated network that is important for maintaining homeostasis through overlapping receptors and key regulatory feedback loops involved in perceived stress, immune responses, and changes in brain activity (Cussotto et al., 2018; Rosin et al., 2021).

The activity of the HPA axis can also impact the composition of the gut microbiota and enteroendocrine cells (EECs), which are distributed throughout the gastrointestinal (GI) tract and are linked to sensory nerve sensitization through direct connections to the enteric nervous system (ENS). (Latorre et al., 2016; Martin et al., 2018; Rosin et al., 2021). Vagal afferent nerves can stimulate the HPA axis, which then in turn signals the adrenal glands to release cortisol (Bonaz et al., 2016). This bidirectional communication underscores the complex interplay between the gut and brain, highlighting how stress and hormonal responses can influence gastrointestinal function and overall homeostasis.

## 5 How can psilocybin impact the gut-brain axis?

**Fig. 2:**
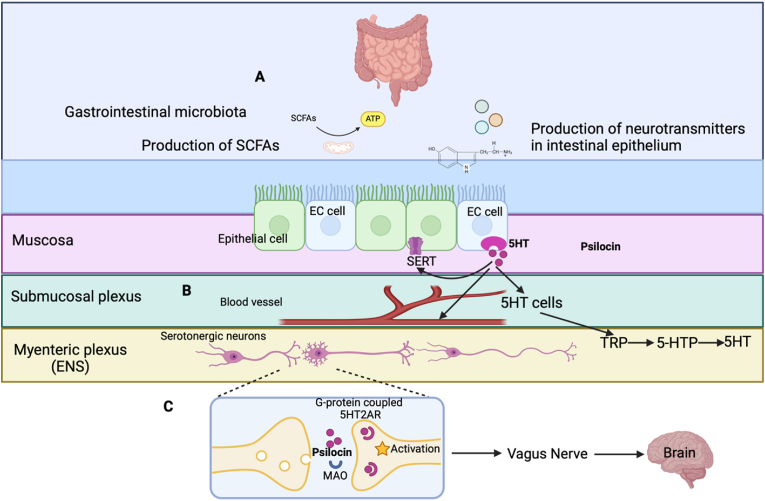
**Schematic representation of pathways in the gut and the role psilocybin plays on the enteric nervous system.** A) Interaction between the microbiota, gut epithelial cell lining, and enteric nervous system act as an axis point for a variety of pathways including the communication between the ENS and CNS. The microbiome gut-brain axis signals the brain through stimulation of the immune system, vagus nerve, microbial metabolites, activation of enteroendocrine cells, enteric nervous system, and the tryptophan-kynurenine pathway. B) As psilocybin is ingested, it is converted into psilocin (a tryptamine), a precursor for serotonin (5-hydroxytryptamine, 5-HT). It is then absorbed in the small intestinal cell lining, submucosal plexus blood stream, where is it able to cross the blood-brain barrier. 5-HT is located in the enterochromaffin cells in the intestinal mucosa and is also synthesized by the microbiota, which produce tryptamines and release 5-HT neurotransmitters, thus activating enteric neurons in the ENS and the vagus nerve endings. C) Further metabolization occurs through monoamine oxidase (MAO) causing acute alteration in the microbiota, responsible for production of neurotransmitters, short chain fatty acids (SFAs), and microbial metabolites. Psilocin, being lipophilic and a 5-HT agonist, permeates through the cell membrane into the neuron and activates the intracellular 5HT_2A_ receptors.

Spore forming bacteria such as Streptococcus spp., Enterococcus spp., Escherichia spp., Lactobacillus plantarum, Klebsiella pneumonia, and *Morganella morganii* are important microbes in the gut that help regulate and promote 5-HT biosynthesis through the mechanism of short-chain fatty acids on colonic EC cells, supplying 5-HT to the mucosa, lumen, and circulating platelets, as shown in Fig. 2 (Reigstad et al., 2015; Yano et al., 2015). Therefore, alterations in the gut microbiota may lead to the dysregulation of serotonergic signaling in the gut and thus potentially eliminating beneficial bacteria, leading to gut dysbiosis and mucosal gut permeability (Reigstad et al., 2015). While 5-HT is a crucial neurotransmitter in the brain, production appears to predominantly occur in the GI neuroendocrine system, which is composed of endocrine cells that reside in the gut mucosa, and the neurons of the ENS (Shajib et al., 2017). Approximately 90–95 % of the body's 5-HT biosynthesis is produced by specific spore forming microbes such as *Clostridium, Lactobacillus, and Bifidobacterium* (Barandouzi et al., 2022; Rusch et al., 2023), challenging the traditional view that centers serotonin's role primarily in the brain (Shine et al., 2022). This critical supply of 5-HT can also act as an immune cell mediator in the mucosa, lumen, and circulating platelets. When acute or chronic intestinal inflammation is triggered, EC cell production increases, resulting in higher levels of 5-HT biosynthesis, where it binds to specific serotonin receptors expressed on various immune cells, including macrophages, dendritic cells, T cells, and mast cells, initiating an innate immune cascade (Koopman et al., 2021). This binding triggers immune cell activation, proliferation, and cytokine production, such as TNF-α, IL-1β, and Interleukin-6 (IL-6) from immune cells, amplifying inflammatory cascades (Koopman et al., 2021; Shajib et al., 2017). Mucosal 5-HT travels to the CNS via the BBB in platelets or up the afferent vagus nerve on 5-HT reuptake transporter proteins in platelets (Fülling et al., 2019; Shajib et al., 2017). 5-HT can interact directly with neuronal, muscle, immune, and epithelial cells (Shajib et al., 2017). Once the physiological function is accomplished, degradation of 5-HT occurs via monoamine oxygenase (MAO) enzyme (Shajib et al., 2017).

Though the mechanisms remain to be fully examined, Kuypers (2019) has hypothesized that two biological mechanisms exist, which together are responsible for the persistent effects of psilocybin. The first being that high doses of psilocybin mainly targets the CNS, and the second is that low consistent doses contribute to sustained effects indirectly via alterations of the composition of the gut microbiota, such as 5-HT production, activation of afferent vagus nerve endings, and production of neurotransmitters. When considering the central role that the microbiome plays in the production of 5-HT, and the impact of psilocybin on serotonergic receptors, more research is required to examine the potential impact of psilocybin on the gut and the subsequent effects on the gut-brain axis.

### 5.1 The tryptophan-kynurenine metabolic pathway

The 5-HT signaling molecule is crucial in the gut-brain axis and is considered to be mainly under the influence of the bacterial microbiota (Cryan et al., 2019). These bacteria produce tryptamines, the precursor for 5-HT and melanin, from the decarboxylation of tryptophan (Trp); an essential amino acid and fundamental precursor for numerous molecules acting at the interface between the host (human) and microbes (Bosi et al., 2020; Fricke et al., 2017). However, Trp is the least abundant amino acid in the body, mainly acquired from the diet, with two catabolic pathways of peripheral Trp metabolism; the kynurenine (Kyn) and 5-HT pathways (Shajib et al., 2017). In the Trp-Kyn pathway, over 95 % of Trp degrades in the body into the active metabolites of quinolinic acid and kynurenic acid, which include proinflammatory, anti-inflammatory oxidative, antioxidative, neurotoxic, neuroprotective, and/or immunologic compounds (Török et al., 2020). Despite being part of the same metabolic pathway, they have distinct biochemical properties, physiological roles, and effects on the nervous system. Quinolinic acid is a neurotoxic compound and an agonist at NMDA receptors, where it can induce excitotoxicity; a process where neurons are damaged and killed by excessive stimulation by neurotransmitters such as glutamate. Quinolinic acid is produced from tryptophan via the intermediate kynurenine, primarily by microglia and macrophages in the brain and other tissues. Conversely, kynurenic acid is considered neuroprotective and acts as an antagonist at NMDA receptors and inhibitor to the kainate and AMPA subtypes of glutamate receptors (Török et al., 2020; Vissel et al., 2001). Its neuroprotective properties suggest a role in protecting the brain from excitotoxicity. Finally, the Kyn enzymatic activities and levels have been shown to directly influence immune and inflammatory responses associated with cancer, autoimmune diseases, sub and chronic inflammation, neurological diseases, and psychiatric disorders (Török et al., 2020).

Trp metabolic dysregulation arises under conditions of stress or gut and brain inflammation, altering axis signaling pathways (Kelly et al., 2023). Stress hormones, such as cortisol, adrenaline, and noradrenaline, as well as cytokines play a significant role in regulating the activity of indoleamine 2,3-dioxygenase (IDO) and tryptophan 2,3-dioxygenase (TDO), two key enzymes in the kynurenine pathway that metabolizes tryptophan (Li et al., 2024). During a stress response, the sympathetic nervous system is upregulated, cortisol is released, which significantly upregulates the expression and activity of TDO. TDO is mainly expressed in the liver and is responsible for the first step in tryptophan catabolism in the kynurenine pathway. The activation of TDO leads to an increased conversion of tryptophan to kynurenine, reducing the level of tryptophan for adequate serotonin synthesis. As well during the stress response, adrenaline and noradrenaline indirectly affect IDO and TDO activity by modulating and amplifying the immune response, thus influencing cytokine production (Török et al., 2020). When looking at IDO activity production and the influence of pro-inflammatory cytokines, such as interferon-gamma (IFN-γ), TNF-α, and IL-6, these strongly induce the expression and activity of IDO (Török et al., 2020). This expression of IDO due to cytokines is a key component of the immune response to infections and inflammatory-related conditions, as IDO-mediated tryptophan depletion can inhibit pathogen growth and modulate immune cell function (Więdłocha et al., 2021). The trp-Kyn pathway may also be influenced by gut microbiota (Więdłocha et al., 2021). If the balance of gut balance is altered or disturbed by various lifestyle impacts, there is potential for increased intestinal permeability of tight junctions (“leaky” gut), which may then lead to increased inflammatory cascades in the submucosal plexus and myenteric plexus and brain via BBB, possibly leading to increased neuroinflammation, gut-brain axis dysfunction, altered neurotransmitter production, and HPA axis overactivation (Teixeira et al., 2022). The resulting inflammation and signaling disruptions may contribute to the development of psychiatric disorders such as anxiety, depression, and cognitive dysfunction (Hawley et al., 2012; Kannangara et al., 2009). Thus, psilocybin might play a potential role in Trp metabolism and optimization of the Kyn pathway associated with gut-brain communication, inflammatory biomarker modulation, and associated neuroplastic changes (Kuypers, 2019).

## 6 Psilocybin, stress, inflammation, and the manifestation of mood disorders

Given that the GI tract is also composed of complex microbiota, which interact with the ENS, balance between optimal intestinal microorganisms, neuroendocrine and immune systems work together to keep the axis in homeostasis (Shanahan et al., 2021). However, dysregulation of these systems in 5-HT signaling and intestinal inflammation have recently been suggested to play a key role in several sub chronic inflammatory-related diseases, including depression and anxiety (Shajib et al., 2017; Terry and Margolis, 2017). As individuals consume and process food, communication between the ENS, PNS, and CNS is vital for maintaining homeostasis (Fülling et al., 2019).

In general, inflammation is defined as a local and systemic repair defense mechanism within the host following an initiation of a physical, chemical, thermal, or biological offense, to restore homeostasis and remove harmful and foreign stimuli (Flanagan and Nichols, 2018). Acute inflammation is a rapid, adaptive, protective response initiated by noxious stimuli, by which the immune system sends inflammatory cells to the site of offense (Harsanyi et al., 2023). In contrast, chronic inflammation is a more prolonged immune response possibly resulting from a variety of systemic impairments such as the immune system failing to eliminate the harmful stimuli, autoimmune disorder, defective cells in charge of moderating inflammation, or inflammatory inducers causing oxidative stress and mitochondrial dysfunction (van Horssen et al., 2019). Considering the pervasive physiological dysregulation manifested as chronic stress and inflammation, it is imperative to adopt an integrative approach when evaluating various psychedelic therapies. This approach should encompass both micro and macro system analyses, given that these therapies exert effects on the peripheral and central nervous systems across the entire body. Understanding psilocybin's effects will require a multisystemic approach to examine the various pathways associated with psilocybin's mechanistic action on the microbiota gut-brain-immune-endocrine axis, to help uncover how psychedelics impact physiological function, may potentially lead to longer term sustained results.

One recent preclinical study investigated how psychedelics influence or help modulate the neuroimmune interactions with fear-related behaviours (Chung et al., 2025). The researchers used a combination of techniques including behavioural assays, molecular and cellular analyses, and neuroimmune cell profiling, to help indicate that astrocytes in the amygdala limit stress-induced fear behaviour through epidermal growth factor receptor. Key findings demonstrated that neuroimmune modulation becomes dysregulated over time due to chronic psychological stress, as supported by an increase of fear behaviour with mice that underwent 18 days of restraint stress as well as an increase in plasma levels of corticosterone and inflammatory cytokines IL-1β, IL-12, TNF and the chemokine MIP2. These results support the argument in this paper by suggesting the potential link between chronic stress, chronic peripheral inflammatory responses, fear behaviour, neuroimmune modulation, and the manifestation of neuropsychiatric disorders. Further, the researchers specifically included that astrocytes are direct targets of corticosterone signaling for chronic stress, linking these inflammatory signals with fear behaviour through glucocorticoid receptor activation and epidermal growth factor receptor upregulation. This specific receptor expression in the amygdala inhibits stress-induced, pro-inflammatory signal transduction cascades, while enabling neuronal-glial crosstalk. These findings support the mechanism of psilocybin for the use of neuroimmune modulation and support for neuropsychiatric disorders (Chung et al., 2025).

**Fig. 3:**
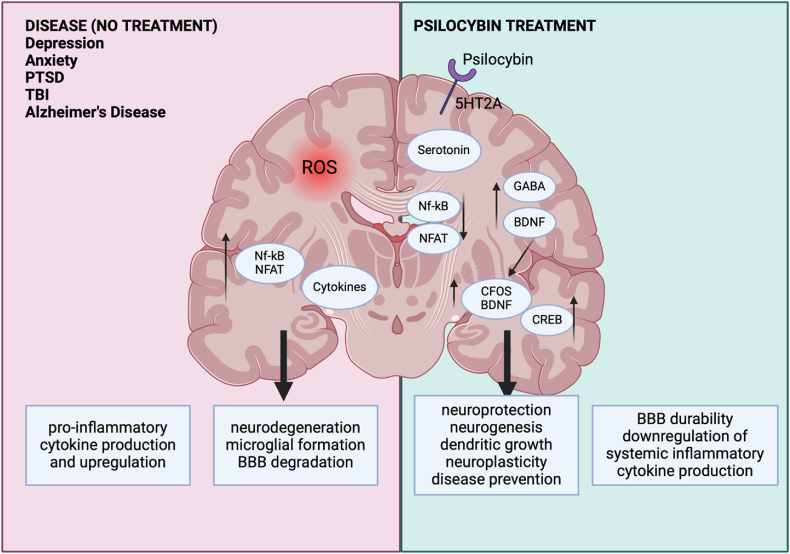
**Psilocybin versus no treatment and the potential associated neuropathological processes.** Neuroplastic effects of psilocybin in relation to psilocybin treatment versus brain without treatment and disease (left). Psilocybin binds to the 5-hydroxytryptamine receptor _2A_ (5-HT_2A_) receptor intra or extracellularly, inducing transcription of brain-derived neurotrophic factor (BDNF), leading to an increase of neuroplasticity, dendritic spine density, neuroprotection, synaptic plasticity and brain region connectivity. Activation of 5-HT_2A_ receptors also bring about protection against oxidative stress by the up regulation of antioxidant genes, such as CREB, a transcription factor known for its role in immune responses, including inhibition of NF-kB activation, transcription regulation of inflammatory cytokines such a TNFα, interleukins, and COX-2. This leads to blood-brain durability and the downregulation of systemic inflammatory cytokine production, enabling greater neural cross communications between various brain regions. Conversely, without treatment of psilocybin, in a diseased state, the brain will exhibit increase reactive oxidate species, an increased transcription of nuclear factor of activated T cells (NFAT) and nuclear factor κB (Nf-kB), which meditates expression of numerous pro-inflammatory genes transcribing cytokines, inflammasomes, resulting in increased microglia formation, a lack of crosstalk communication between brain regions, thus neurodegeneration and persistent disease.

All this to say, to date, little to no research has been conducted on psychedelics and the gut-brain axis, but several studies have demonstrated a link between gut dysbiosis, gut permeability (i.e., “leaky gut”), inflammation, and the prevalence of numerous mood disorders (Camilleri, 2019; Limbana et al., 2020; Shajib et al., 2017). One notable review discussed disruption in the intestinal barrier and dysbiosis leading to increased intestinal permeability of bacterial products, leading to release of pro-inflammatory cytokines, such as IL-1β, TNF-α, IL-6, and reactive oxidative species, and ultimately to increased permeability of the BBB, neuroinflammation, thus an increased probability in mood disorders (Fig. 3) (Rudzki and Maes, 2020). It has been demonstrated that food antigens or insults interacting with the gut microbiome – such as stress, sugar, or environmental toxins – can result in gut dysbiosis, triggering a leaky gut (Camilleri, 2019). Several studies have found an association between increased levels of peripheral cytokines, as a result of leaky gut, and increased rates of depression and anxiety (Chapman and Graham, 2020; Harsanyi et al., 2023). Certain cytokines can act on vagus nerve afferents, leading to changes in neuroinflammation, including the production of reactive oxidative species (ROS), activation of nuclear factor of activated T-cells (NFAT) and NF-kB pathways, increased enzyme IDO activity, involvement of glial cells, neurodegeneration, and degradation of the BBB (see Fig. 3) (Fülling et al., 2019; Julio-Pieper et al., 2014; Liu et al., 2020; Rudzki and Maes, 2020). Although still very novel in preclinical psychedelic research, psilocybin and other serotonergic psychedelics have demonstrated immunomodulatory effects via regulation of TNF-α meditated inflammation (Yu et al., 2008). Interestingly, Yu et al. (2008) looked at the psychedelic and 5-HT_2A_ receptor agonist (R)-2,4-dimethoxy-4-iodoamphetamine [DOI] and its modulatory effects of TNF-α levels in smooth muscle cells. The researchers found that 5-HT_2A_ receptor activation strongly inhibited TNF-α-mediated pro-inflammatory gene expression of intracellular adhesion molecule 1 (ICAM-1), vascular adhesion molecule 1 (VCAM-1), and IL-6, molecules crucial for immune cell recruitment and inflammatory cascades. DOI also blocked nitric-oxide synthase activity and nuclear translocation of NF-κB, further demonstrating the potent pleiotropic anti-inflammatory outcome in smooth muscle cells. Furthermore, 5-HT_2B_ and 5-HT_2C_ receptor agonists did not subpress TNF-α-meditated inflammatory cascades, further indicating these regulatory effects are 5-HT_2A_ specific. Though tested on smooth muscle cells, the findings suggest that 5-HT_2A_ receptor activation by compounds like DOI can reduce inflammation through multiple pathways, including inhibition of inflammatory gene expression, reduction in oxidative stress, and suppression of key transcription factors. We propose that these anti-inflammatory properties could be relevant in treating conditions characterized by chronic inflammation, such as cardiovascular diseases, autoimmune disorders, and possibly neuroinflammatory diseases. Though DOI slightly differs in structure, it contains a similar mechanistic activation of 5-HT_2A_ receptors. Therefore, these compelling findings suggest the opportunity to further explore the potential of psilocybin as an immuno-modulator of inflammatory pathways, providing a mechanistic basis for their broad therapeutic applications beyond their neurological effects.

## 7 Dosing psilocybin

Research into the therapeutic potential of psilocybin has been primarily conducted using administration of psilocybin at high doses, often referred to as “macrodoses” (Davis et al., 2021; Lowe et al., 2021). From a safety and toxicological perspective, psilocybin is well tolerated with low toxicity (Pedicini and Cordner, 2023). In fact, a lethal dose has not been established in humans, but the known LD_50_ of intravenous psilocybin in rats (280 mg/kg) is more than 700 times the typical clinical study high dose (20–30 mg) for an average of 70 kg bodyweight (Schlag et al., 2022). In other words, the lethal dose has been estimated to be 1000 times the effective dosing (Johnson et al., 2018). Other studies have use fixed dose approaches rather than weight-adjusted doses (Garcia-Romeu et al., 2021; Grob et al., 2011; Johnson et al., 2014). To determine whether bodyweight impacts the effects of psilocybin, researchers administered participants with either a weight-adjusted dose or a fixed dose of psilocybin (Garcia-Romeu et al., 2021). After assessing therapeutic outcomes, demographic variables, and sex differences, they found no significant differences between either psilocybin administration method, suggesting that fixed doses should be considered in trials moving forward, with the exception of extreme cases of gastrointestinal conditions such as disordered eating, severe obesity, and inflammatory bowel disease.

In recent years, the concept of “microdosing”, which entails the repeated consumption of a very low concentration of a psychedelic compound, such as lysergic acid diethylamide (LSD) or psilocybin-containing mushrooms, has gained popularity in research and personal use (Rootman et al., 2021, 2022a). Importantly, microdosing produces effects that are sub-perceptive and sub-hallucinogenic (Cameron et al., 2020). A microdose is generally one tenth of a typical high dose, and the practice of microdosing typically follows a dosing scheme (Anderson et al., 2019b; Fadiman and Korb, 2019; Rosenbaum et al., 2020a). The intentions behind microdosing vary but seem to primarily be a desire to improve one's daily sense of well-being and to enhance cognitive, creative, or emotional processes (Anderson et al., 2019b). Surveys of self-reported psilocybin microdosing regimes have varied in protocols (Cavanna et al., 2022), but the average dosing in humans includes oral self-administration of 0.1–0.3 g of dried psilocybin-containing mushrooms, three to five times per week (Polito and Stevenson, 2019; Rootman et al., 2021; Rosenbaum et al., 2020b). Preclinical microdosing studies are sparse; however, the existing studies include doses of between 0.025 and 0.1 mg/kg in rodent models (Cameron et al., 2019; Horsley et al., 2018). Prefatory qualitative microdosing research in recent years has indicated an enhancement of mood, cognition psychomotor functioning, and general well-being (Anderson et al., 2019a, 2019b; Rootman et al., 2022b; Rosenbaum et al., 2020a, 2020b). Reductions in stress, fatigue, depression, and anxiety have also been observed in a handful of cross-sectional survey studies (Cameron et al., 2020; Kaertner et al., 2021; Lea et al., 2020; Polito and Stevenson, 2019). With use of questionnaires, interviews, focus groups, and anecdotal data collection, results still suggest greater dose controlled empirical research is required to support the impacts of microdosing on mental health, well-being, and their neural underpinnings. Despite the need for exploratory naturalistic research and metrics, this preliminary research does pose limitations of interpretation bias and unexpected variability. Reductions in stress, fatigue, depression, and anxiety have also been observed in a handful of cross-sectional survey studies (Cameron et al., 2020; Kaertner et al., 2021; Lea et al., 2020; Polito and Stevenson, 2019). With use of questionnaires, interviews, focus groups, and anecdotal data collection, results still suggest greater dose controlled empirical research is required to support the impacts of microdosing on mental health, well-being, and their neural underpinnings. Despite the need for exploratory naturalistic research and metrics, this preliminary research does pose limitations of interpretation bias and unexpected variability.

There is a growing acknowledgement around the importance of accounting for sex differences in preclinical and clinical psychedelic research, when considering both high and low dose psilocybin administration. For example, one recent study specifically probed for sex-specific effects on Sprague-Dawley rats after a single high dose of subcutaneous psilocin (2 mg/kg) and assessed activity on the central nucleus of the amygdala (CeA) using fiber photometry paired with an aversive air-puff stimulus, and tracked behavioural responses (Effinger et al., 2023). Researchers reported CeA reactivity in both sexes post psilocin administration. However, psilocin yielded time-dependent and sex-specific changes in CeA reactivity 28 days following psilocybin administration, with a decrease found only in males. This study further demonstrates potential sex-specific, time-dependent, CeA activity and reactivity changes, and behavioural responses to aversive stimuli from a single-dose administration of psilocin via subcutaneous injection in male and female Sprague-Dawley rats. Extensive research efforts are required to uncover sexual dimorphisms in neurochemical signaling pathways involved in psilocybin's action, including receptor activation, hormonal activity, neurotransmitter synthesis, release, and removal, and functional brain connectivity. Understanding proper psychedelic dosing is critical for determining personalized therapeutic thresholds that optimize neuroimmune modulation without unwanted side effects for varying neuropsychiatric disorders, while also considering sex differences.

## 8 Summary and conclusions

Understanding the complex mechanisms of psilocybin at both high and low doses offers a valuable framework for exploring its clinical potential. We believe these topics presented in this paper offer a necessary mechanistic framework that justifies further therapeutic exploration of psilocybin in addressing stress-induced disorders through gut-brain-immune modulations. By examining its effects on peripheral and central biomarkers, researchers can better predict its relevance in addressing disease states linked to serotonergic dysregulation and inflammatory pathways. As highlighted in this review, gut dysbiosis, increased intestinal permeability, and gut inflammation directly contribute to mood disorders and inflammation-related diseases. Numerous studies have demonstrated that chronic systemic inflammation, coupled with elevated cortisol levels, activates the HPA axis and sympathetic nervous system (SNS) while suppressing parasympathetic activity. This imbalance predisposes individuals to stress, anxiety, depression, and other mood disorders, underscoring the need for interventions targeting both neuroendocrine and immune pathways. High and low dose psilocybin hold a variety of applications when looking at targeting cascades within the gut-brain-endocrine-immune systems. It is hypothesized that low doses of psilocybin may act in a more gradual manner by indirectly impacting the CNS through interaction with active microbiome metabolites, 5-HT and the Trp-Kyn pathway, while high doses of psilocybin may be impacting the CNS directly on a more rapid level, by activating 5-HT_2A_ receptors, suppressing the NF-κB pathway, regulating oxidative stress, thereby lowering neuroinflammation and enhancing BDNF transcription factors. The gut-brain axis is an influential matrix where the bloodstream, neuroimmune, neuroendocrine, and neurotransmission signal the ENS to the CNS via vagal activation. Therefore, by taking a more multi-system mechanistic approach to future studies, this may lead to a more comprehensive understanding of psilocybin's therapeutic potential when weaving in the bidirectional gut-brain axis pathway, leading to clinical optimization and even disease prevention.

## 9 Future research

Future research models should look at the gut-brain axis as an impactful mechanism of psilocybin and subsequent serotonergic cascades via microbiome assessments, inflammatory blood biomarkers, central markers of brain activity, consideration of sex differences, and behavioural testing in preclinical and clinical populations. Having greater preclinical and clinical studies that examine the gut's interaction with psilocybin, will also lead to a greater mechanistic understanding of how psilocybin affects the brain, behaviour, and mood. Furthermore, translational models are suggested to incorporate lifestyle assessments including diet, exercise, sleep, and alcohol or drug use, for a greater comprehensive understanding in the overlapping relationship of psilocybin therapy on lifestyle behavioural changes and the GBA axis. Therefore, gathering additional data on these multi-faceted pathways could provide valuable insights for practitioners and patients, enabling more effective strategies for personalized therapeutic preparation, dosing, and integration of high- and low-dose psilocybin therapeutics. This tailored approach aims to optimize and sustain outcomes, ultimately enhancing the quality of life.

## CRediT authorship contribution statement

**Alanna Kit:** Writing – review & editing, Writing – original draft, Investigation, Conceptualization. **Kate Conway:** Writing – original draft, Investigation, Formal analysis, Conceptualization. **Savannah Makarowski:** Writing – review & editing, Investigation. **Grace O'Regan:** Formal analysis, Visualization, Writing – review & editing. **Josh Allen:** Writing – review & editing, Validation, Investigation. **Sandy R. Shultz:** Writing – review & editing, Supervision, Investigation. **Tamara S. Bodnar:** Writing – review & editing, Writing – original draft, Supervision, Investigation. **Brian R. Christie:** Writing – review & editing, Writing – original draft, Supervision, Software, Resources, Project administration, Methodology, Investigation, Funding acquisition, Data curation, Conceptualization.

## Funding

This work was supported by a grant from NSERC to BRC.

## Declaration of competing interest

The authors declare that they have no known competing financial interests or personal relationships that could have appeared to influence the work reported in this paper.
